# Refocusing on nature: the role of nature reintegration in environmental, mental, and societal wellbeing

**DOI:** 10.3389/fpsyg.2024.1351759

**Published:** 2024-02-27

**Authors:** Royce L. Willis, Eric Brymer, Vinathe Sharma-Brymer, Matthew Leach

**Affiliations:** ^1^Faculty of Health, Southern Cross University, Lismore, NSW, Australia; ^2^Manna Institute, University of New England, Armidale, NSW, Australia; ^3^School of Law and Society, University of the Sunshine Coast, Maroochydore, QLD, Australia

**Keywords:** climate change, environmental education, environmental policy, green space, mental health, human-nature connection, nature-based interventions, pro-environmental behavior

## Abstract

This perspective paper investigates the dynamic interplay between wealth, materialism, environmental degradation, and mental health amid escalating challenges of climate change. The paper critically examines how affluence, often a buffer against climate impacts, paradoxically leads to higher consumption and carbon footprints, exacerbating environmental problems. A societal emphasis on materialism contributes to an estrangement from nature, with significant implications for mental health and environmental sustainability. The paper proposes a fundamental shift in addressing these intertwined challenges through reintegration with nature. The paper recommends integrating urban planning, education, mental health, and community engagement strategies to build a sustainable, mentally resilient society more integrated with nature. This approach, supported by future research directions, aims to create a more balanced, environmentally conscious, and mentally healthy world.

## 1 Introduction

Climate change introduces a host of ecological disturbances and contributes to an increasing physiological and psychological toll (Ripple et al., 2020). This paper explores nature reintegration as a potential pathway toward a more favorable relationship between humans and their environment. Four key dimensions will be used to support its central thesis: (1) the paradox of wealth and environmental impact; (2) detachment from nature; (3) pro-environmental behavior and mental health; and (4) the need for nature reintegration.

## 2 Foundational concepts

### 2.1 The paradox of wealth and environmental impact

Environmental stewardship and a strong human-nature relationship are vital in the context of global environmental challenges. Environmental stewardship involves collective actions by individuals, groups, or networks to responsibly protect, manage, and utilize the environment for environmental and social good (Bennett et al., 2018). This includes activities like conservation efforts, reforestation, pollution reduction, and sustainable resource use, implemented across various scales. Such stewardship will likely play an increasingly critical role in addressing the pressing issue of climate change.

Contemporary societies strongly emphasize wealth growth and materialism, often overshadowing the importance of the human-nature relationship and its impact on environmental stewardship and nature integration. Financial wealth can act as a temporary buffer against the impacts of climate change, with financially affluent individuals and nations often possessing the resources to adapt to such change (Asfaw et al., 2019; Nauges et al., 2021). Adaptations might include installing air-conditioning or building flood defenses, which reduce immediate environmental impact and psychological stressors. However, this financial affluence is often linked with higher levels of consumption and, consequently, a larger carbon footprint (Mi et al., 2020; Barros and Wilk, 2021; Chancel, 2022), presenting a paradox. The very groups that contribute most significantly to climate change often have the means to shield themselves from its direct effects. This dichotomy highlights the need for a broader and more inclusive approach to environmental stewardship, transcending economic status and focusing on sustainable practices at all levels of society.

The prevalent focus on financial wealth and material comfort in modern societies and subsequent ignorance of the human-nature relationship has implications for pro-environmental behavior. Materialistic values have been shown to negatively impact such behavior (Ahlström et al., 2020; Kelly et al., 2021), leading to increased environmental degradation. For example, populations in the lower 50% income bracket in the USA and some European countries are close to or already achieving 2030 per-capita emission targets (Chancel, 2022). By contrast, populations in the top 10% income bracket must reduce emissions by over 80% to meet these targets. Wealth may also contribute to an estrangement from nature, as material comforts and unsustainable consumption displace the desire for natural experiences (Bogert et al., 2022). A societal shift toward valuing the human-nature relationship alongside material success could benefit both the environment and human wellbeing.

### 2.2 Detachment from nature

In financially affluent Western societies, there is increasing isolation from the natural environment, with individuals experiencing nature less frequently and in lesser quality (Cazalis et al., 2023). This estrangement, a phenomenon where humans view themselves as separate from or outside of nature, has been instrumental in environmental neglect and degradation (Lokhorst et al., 2014; Marczak and Sorokowski, 2018; Tam, 2019; Bogert et al., 2022). Such neglect has contributed to today’s environmental crisis, exacerbating climate change, and detrimentally affecting mental health (Hayward and Ayeb-Karlsson, 2021; Thoma et al., 2021). The escalation of climate change-related events, such as severe weather events and bushfires, is directly linked to a range of mental health disorders, including anxiety, depression, PTSD, eco-anxiety, and poor mental wellbeing (Dodgen et al., 2016; Clayton et al., 2017; Cianconi et al., 2020; Palinkas and Wong, 2020; Hayward and Ayeb-Karlsson, 2021; Thoma et al., 2021). These mental health implications, especially among vulnerable populations, indicate that climate change is a substantial public health concern (Verplanken et al., 2020; Parry et al., 2022).

The estrangement from nature is not an isolated phenomenon; it is linked to societal values, consumerist behaviors, and policy decisions (Everard et al., 2016; Sharma and Jha, 2017; Dong et al., 2020; Molinario et al., 2020; Bogert et al., 2022). The degradation of the environment negatively impacts human mental health (Dodgen et al., 2016; Cianconi et al., 2020), with poor mental health adversely affecting decision-making and behavior (Kung et al., 2018). In other words, the relationship between the degradation of the environment and mental health represents a vicious downward spiral.

Biophobia contributes to the detachment from nature in contemporary societies (Soga et al., 2023). This concept, contrasting with the biophilia hypothesis of an innate human affinity for nature, refers to negative emotions like fear and disgust toward natural elements (Simaika and Samways, 2010). Biophobia can present as a specific fear, for example, of insects, spiders, or snakes (Soga and Gaston, 2022a). It is thought to be more prevalent and intense in urbanized societies, with exposure to negative information about nature, parental influences, and media portrayals potentially intensifying these negative emotions (Zhang et al., 2014; Soga et al., 2020; Fukano and Soga, 2021; Vanderstock et al., 2022). The unpleasant feelings associated with biophobia (Bhaumik et al., 2020) can lead to avoidance of nature and, consequently, further disconnection from the natural environment. This detachment impacts biodiversity conservation (Sumner et al., 2018; Castillo-Huitrón et al., 2020) and deprives individuals of the mental health benefits provided by nature (Buxton et al., 2021; Maes et al., 2021; Marselle et al., 2021; Soga and Gaston, 2022b).

Reinvigorating a sense of connection or being part of nature is not merely a return to sustainable living but a fundamental shift in the approach to mental health and environmental policy. It is also a fundamental shift in how we understand psychology and, as a result, mental health. The reinvigoration of the human-nature relationship—essential to addressing the intertwined challenges of mental health and environmental sustainability—is a foundation for future research and policy initiatives. Reinvigoration promises to foster pro-environmental behavior and catalyze a shift toward environmentally responsible policies, creating a virtuous cycle that enhances both the environment and human wellbeing.

### 2.3 Pro-environmental behavior and mental health

Eco-anxiety can occur in response to ecological crises and climate change, characterized by worry, existential distress, and feelings of uncertainty, lack of control, and being overwhelmed (Panu, 2020). It encompasses a spectrum of reactions, from mild anxiety to severe impacts similar to depression and PTSD. While it can be paralyzing, eco-anxiety may also motivate individuals to reassess their lifestyles and environmental behaviors. Although some authorities advocate the use of psychological therapies for the management of eco-anxiety, such as building resilience and hope (Usher et al., 2019), eco-anxiety is not irrational. That is, there are often sound reasons for people to be concerned about climate change. Psychological therapies to build resilience to climate change and environmental issues may also have an untoward effect by reducing pro-environmental behaviors (Shao and Yu, 2023).

Engaging in pro-environmental behaviors offers dual benefits. Such behaviors contribute to environmental sustainability by reducing waste, conserving resources, and mitigating the impacts of climate change. There is also evidence of a positive association between pro-environmental behaviors and mental wellbeing (Zawadzki et al., 2020), possibly by building resilience, which mitigates the psychological stressors induced by climate change. This association is significant given the high prevalence and economic burden of mental health disorders globally (Arias et al., 2022).

Pro-environmental behaviors can also serve as a coping mechanism. By taking actionable steps to address environmental issues, individuals may experience a sense of agency and control, which is beneficial for mental health (Innocenti et al., 2023). Therefore, pro-environmental behavior transcends ethical obligation, serving as a pragmatic strategy to enhance environmental and mental health outcomes.

### 2.4 The need for nature reintegration

Intrinsic motivation for pro-environmental behavior, driven by a fundamental understanding that humans are part of nature, can lead to broader adoption of sustainable practices and support for environmentally friendly policies (Maki et al., 2019; Liu et al., 2022). Whether we acknowledge it or not, humans rely on nature to survive. From habitat, food production, climate regulation, and experiential mental health effects, nature provides human with the essential ingredients to support their existence (Brauman et al., 2020). Unfortunately, many ecosystems that provide these essential ingredients are being neglected or destroyed. So, although humans are heavily dependent on nature, they are often estranged from nature, sometimes to the extent of ignoring or being oblivious to the importance of nature. A reintegration with nature offers multiple benefits: highlighting nature’s importance for human existence, fostering mental resilience against climate-induced stressors, and catalyzing pro-environmental behaviors. These outcomes are critical to pursuing a more sustainable and mentally resilient society.

Integrating insights from environmental and ecological psychology provides a robust theoretical basis for understanding the human-nature connection. Environmental psychology emphasizes the role of natural environments in influencing human behavior and wellbeing, including how green spaces enhance mental health, reduce stress, and support cognitive function (Kaplan and Kaplan, 1989). Ecological psychology complements environmental psychology by focusing on the reciprocal relationship between individuals and their environment, where behavior is shaped by the perception of environmental affordances (Gibson, 1979). Environmental psychology investigates interactions between humans and their surroundings, whereas ecopsychology focuses on the emotional ties and dynamics between human psychology and nature, seeking to promote a sustainable and balanced relationship (Doherty, 2010). Together, these perspectives underscore the importance of human connection with nature in promoting mental health, sustainable behaviors, and resilience against environmental challenges. This approach demonstrates how natural environments provide essential psychological benefits and actively shape human interactions with the world, fostering a deeper, more meaningful engagement with nature.

Acknowledging the significant role of environmental education in promoting a deeper connection between individuals and the natural world is crucial. Environmental education fosters pro-environmental behaviors and enhances subjective wellbeing by nurturing intrinsic motivation, increasing environmental hope, and strengthening nature connectedness (Kerret et al., 2016, 2020). Through targeted educational programs, hope and wellbeing can be boosted, improving knowledge, awareness, and encouraging collective action, fostering a strong sense of community and place attachment.

Facilitating a reintegration with nature goes beyond simple outdoor activities; it involves a deeper emotional, psychological, and philosophical engagement with the natural world (Ives et al., 2018). This form of engagement is essential to promoting tangible societal changes in sustainability (Abson et al., 2017; Ives et al., 2018). Advocating reintegration with nature does not suggest a return to a primitive, sustainable way of living but rather a fundamental shift in our approach to mental health and environmental policy. This approach is beneficial and essential, serving as a cornerstone for future research and policy initiatives addressing mental health and environmental sustainability challenges.

## 3 Framework for a more sustainable, mentally resilient, and nature-connected society

**Figure 1:**
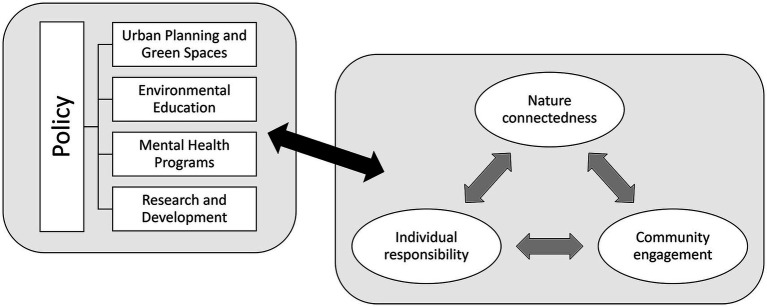
Framework for a more sustainable, mentally resilient, and nature-integrated society.

The following section translates the previously discussed concepts into practical strategies. The strategies can be divided into six interconnected themes: Urban Planning and Green Spaces, Environmental Education, Mental Health Programs, Research and Development, Individual Responsibility, and Community Engagement. Each theme fosters nature connectedness, enhances community involvement, and promotes individual responsibility toward environmental and mental health. It is envisioned that these themes will form a framework to support a more sustainable, mentally resilient, and nature-connected society. Figure 1 provides a visual representation of the framework.

1 **Urban Planning and Green Spaces**:

- Promote nature connectedness by creating accessible green spaces that allow individuals and communities to engage directly with a biodiverse nature.
- Foster community engagement and individual responsibility through stewardship and care of these green spaces.
- Utilize green spaces as living laboratories for research on urban ecology and the benefits of nature connectedness.

2 **Environmental Education**:

- Promote nature connectedness with experiential learning in local green, biodiverse spaces.
- Increase individual responsibility by teaching the importance of being part of nature alongside environmental issues and sustainable practices.
- Strengthen community engagement through educational initiatives encouraging nature experiences and fostering a deeper understanding of the human-nature relationship.
- Build an evidence-base linking educational initiatives with changes in environmental attitudes and behaviors.

3 **Mental Health Programs**:

- Utilize nature-based and solution-based therapies to enhance individual responsibility for mental health through experiences of nature reintegration and a sense of agency and control.
- Integrate shared nature experiences into community programs that promote communal wellbeing and resilience.
- Generate evidence about the therapeutic effects of nature on mental health and overall wellbeing.

4 **Research and Development**:

- Utilize evidence supporting the benefits of nature connectedness to inform urban planning and green space development.
- Support the creation of environmental education curricula that emphasize the human-nature relationship.
- Enhance community engagement and individual responsibility by providing insights into the role of nature in promoting mental health and societal wellbeing.

5 **Individual Responsibility**:

- Provide individuals with opportunities to seek out and create nature connectedness through education and the provision of biodiverse green spaces.
- Drive demand for urban planning that facilitates nature experiences and supports environmental education initiatives focusing on nature connectedness.
- Influence the development of mental health programs that incorporate nature-based approaches.

6 **Community Engagement**:

- Support urban planning initiatives that create communal biodiverse green spaces fostering nature connectedness.
- Facilitate engagement in environmental education programs that bring communities closer to nature.
- Promote the benefits of mental health programs that leverage the power of communal experiences in nature to support collective wellbeing.

## 4 Future research directions

The proposed framework advocates for comprehensive research that interconnects Urban Planning and Green Spaces, Environmental Education, Mental Health Programs, Research and Development, Individual Responsibility, and Community Engagement. For instance, future research is needed to better understand the mechanisms through which nature reintegration can mitigate mental health issues caused by climate change. There is also a need to identify strategies that effectively promote pro-environmental behaviors through urban planning and community engagement.

## 5 Conclusion

This perspective paper has explored the complex interplay between wealth and materialism, environmental impact, and mental health in the context of accelerating climate change. While financial wealth can provide a means to adapt to environmental changes, it correlates with increased consumption and a larger carbon footprint, exacerbating environmental issues. This trend is intensified by a societal focus on materialism, often at the cost of becoming estranged from nature. The paper advocates for restoring human connection with nature, not necessarily as a return to simpler living, but as a vital shift in the approach to mental health, environmental policy, and societal values: a reintegration of biodiverse nature into human society. Emphasizing nature connection over materialistic pursuits could lead to more sustainable, mentally resilient communities. This approach, supported by the proposed strategies and future research directions, offers a path toward a more balanced and environmentally conscious society.

## Data availability statement

The original contributions presented in the study are included in the article, further inquiries can be directed to the corresponding author.

## Author contributions

RW: Conceptualization, Investigation, Writing – original draft, Writing – review & editing. EB: Conceptualization, Writing – review & editing. VS-B: Writing – review & editing. ML: Writing – review & editing.
